# Indoleamine 2,3-Dioxygenase (IDO) Enzyme Links Innate Immunity and Altered T-Cell Differentiation in Non-ST Segment Elevation Acute Coronary Syndrome

**DOI:** 10.3390/ijms19010063

**Published:** 2017-12-26

**Authors:** Chiara Zara, Anna Severino, Davide Flego, Aureliano Ruggio, Daniela Pedicino, Ada Francesca Giglio, Francesco Trotta, Claudia Lucci, Domenico D’Amario, Ramona Vinci, Eugenia Pisano, Giulio La Rosa, Luigi Marzio Biasucci, Filippo Crea, Giovanna Liuzzo

**Affiliations:** Department of Cardiovascular and Thoracic Sciences, Catholic University of the Sacred Heart, Fondazione Policlinico Universitario A. Gemelli, 8, 00168 Largo A. Gemelli, Italy; chiara.zara11@gmail.com (C.Z.); annaseverino71@gmail.com (A.S.); davideflego@gmail.com (D.F.); aurelianoruggio@gmail.com (A.R.); danialla@gmail.com (D.P.); ada.giglio@gmail.com (A.F.G.); fr.trotta@hotmail.it (F.T.); claudia.lucci87@gmail.com (C.L.); domenico.damario@gmail.com (D.D.); ramona.vinci@hotmail.it (R.V.); eugenia.pisano@libero.it (E.P.); giulio.larosa90@gmail.com (G.L.R.); lmbiasucci@virgilio.it (L.M.B.); filippo.crea@unicatt.it (F.C.)

**Keywords:** acute coronary syndromes, immune system, IDO, myeloid derived dendritic cells, T-cell differentiation, personalized medicine

## Abstract

Atherosclerosis is a chronic inflammatory disease characterized by a complex interplay between innate and adaptive immunity. Dendritic cells (DCs) play a key role in T-cell activation and regulation by promoting a tolerogenic environment through the expression of the immunosuppressive enzyme indoleamine 2,3-dioxygenase (IDO), an intracellular enzyme involved in tryptophan catabolism. IDO expression and activity was analyzed in monocytes derived DCs (MDDCs) from non-ST segment elevation myocardial infarction (NSTEMI) patients, stable angina (SA) patients and healthy controls (HC) by real-time quantitative polymerase chain reaction (RT-qPCR) before and after in vitro maturation with lipopolysaccharide (LPS). The amount of tryptophan catabolite; kynurenine; was evaluated in the culture supernatants of mature-MDDCs by ELISA assay. Autologous mixed lymphocyte reaction (MLR) between mature-MDDCs and naïve T-cells was carried out to study the differentiation towards T-helper 1 (Th1) and induced regulatory T-cells (iTreg). Analysis of IDO mRNA transcripts in mature-MDDCs revealed a significant reduction in cells isolated from NSTEMI (625.0 ± 128.2; mean ± SEM) as compared with those from SA (958.5 ± 218.3; *p* = 0.041) and from HC (1183.6 ± 231.6; *p* = 0.034). Furthermore; the concentration of kynurenine was lower in NSTEMI patients (2.78 ± 0.2) and SA (2.98 ± 0.25) as compared with HC (5.1 ± 0.69 ng/mL; *p* = 0.002 and *p* = 0.016; respectively). When IDO-competent mature-MDDCs were co-cultured with allogeneic naïve T-cells, the ratio between the percentage of generated Th1 and iTreg was higher in NSTEMI (4.4 ± 2.9) than in SA (1.8 ± 0.6; *p* = 0.056) and HC (0.9 ± 0.3; *p* = 0.008). In NSTEMI, the tolerogenic mechanism of the immune response related to IDO production by activated MDDCs is altered, supporting their role in T-cell dysregulation.

## 1. Introduction

Although the early outcome of acute coronary syndromes (ACS) has recently considerably improved, cardiovascular diseases still represent the leading cause of mortality worldwide.

An adaptive immunity imbalance, mostly involving CD4^+^ T-cell subsets, has been documented among ACS with systemic evidence of inflammation [1]. In particular, regulatory T-cells (Treg) are reduced, while effector T helper (Th)-1 and Th17 lymphocytes are expanded [2,3,4]. Moreover, T-cells from ACS patients show T-cell receptor (TCR) signaling abnormalities leading to enhanced immune response and altered T helper differentiation [5,6].

Atherosclerotic lesions also contain abundant innate immune cells, including antigen presenting cells (APCs) that take part in initiation, progression and destabilization of the atherosclerotic plaque [7]. Among these, dendritic cells (DCs) are a heterogeneous pool of professional APCs with the ability to sense a wide array of stimuli and translate innate into adaptive immunity by directing an appropriate T-cell response [8,9].

DCs’ functional plasticity enables them to induce both tolerance and immune activation, depending on incoming signals [9,10,11]. Therefore, these cells can contribute to atherosclerosis in both ways, by supporting proatherogenic vascular inflammation and by suppressing T-cell response via induction of tolerogenic properties [12]. Once activated, DCs can promote a tolerogenic environment through the expression of the immunosuppressive enzyme indoleamine 2,3-dioxygenase (IDO) [13].

IDO is an intracellular enzyme involved in tryptophan catabolism pathway. This enzyme catabolizes the essential amino acid tryptophan (l-Trp) into *N*-formylkynurenine, which is in turn rapidly converted by *formidase* to the stable metabolite L-kynurenine. Kynurenine is subsequently metabolized to downstream bioactive molecules [14]. IDO expression is induced by inflammatory mediators, such as IFN-γ [15], even if an IFN-γ independent pathway of activation has been described [16].

IDO-dependent T-cell suppression is mediated by direct effects on T-cells (through tryptophan depletion or by downstream toxic metabolites), indirect effects (through functional alteration of the DCs) and by linked suppression of neighboring IDO-APCs [13]. Different cell types, in addition to DCs, express IDO, such as leucocytes, endothelial cells (ECs), macrophages and vascular smooth muscle cells (VSMCs), all of them abundantly present in the artery wall.

IDO and IDO-induced tryptophan degradation-dependent pathways might have a key role in cardiovascular diseases [17]. In the Tampere Vascular Study, increased IDO expression was observed in the macrophage-rich cores of human advanced atherosclerotic plaques [18] and, more recently, in patients with stable angina pectoris, elevated plasma kynurenine levels have been demonstrated to predict increased risk of acute myocardial infarction [19].

In the present study, we used an ex vivo model to investigate the role of IDO-competent DCs in the cross-talk between innate and adaptive immunity in non-ST segment elevation myocardial infarction (NSTEMI) patients. We studied markers of monocyte-derived DCs (MDDC) maturation, the expression of IDO and the kynurenine pathway in MDDCs from patients presenting with NSTEMI, stable angina (SA) and healthy controls (HC) after stimulation with lipopolysaccharide (LPS). Finally, in the same groups of study, we performed co-culture experiments between autologous LPS-maturated MDDCs and isolated naïve CD4^+^ T-cells to assess IDO-dependent T-cell differentiation in NSTEMI.

We observed an alteration in MDDCs’ maturation and a reduced expression of the immunomodulatory enzyme IDO in NSTEMI patients. In the same group we also observed an increased naïve CD4^+^ T-cell differentiation towards aggressive effector Th1 lymphocytes after polarization with LPS-maturated MDDCs, whereas there were no differences in T-cell differentiation after the T-cell receptor (TCR) stimulation and the exposure to cytokine mixture. Our study supports the role of IDO and MDDCs in NSTEMI T-cell dysregulation.

## 2. Results

### 2.1. MDDC Maturation was Altered in NSTEMI Patients

**Figure 1:**
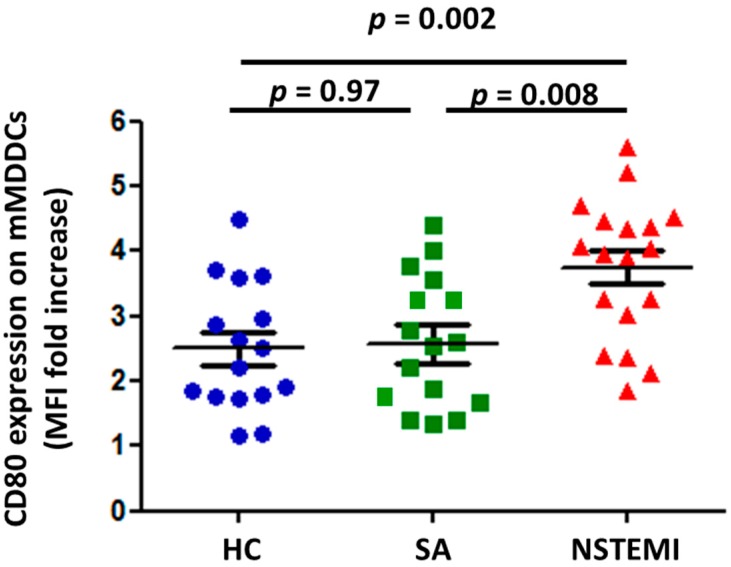
Altered monocytes derived dendritic cells (MDDC) maturation in non-ST segment elevation myocardial infarction (NSTEMI) patients. Monocytes from 18 NSTEMI, 16 SA and 16 HC were differentiated in vitro for 6 days to generate immature MDDCs (iMDDCs). For MDDCs activation (mMDDCs), iMDDCs were exposed to 1 ng/mL LPS for 24 h (Figure S1). CD80 expression on mMDDCs was higher in NSTEMI patients compared with SA and HC (*p* for trend = 0.003). Data are presented as single dot plots and means ± SEM of MFI fold increased respect to iMDDCs. *MDDCs* = myeloid derived dendritic cells; *SEM* = standard error of mean; *MFI* = mean fluorescence intensity.

Peripheral blood monocytes were differentiated into immature MDDCs (iMDDCs) as described in *Methods*. Stimulation for 24 h with LPS induced maturation of MDDCs (mMDDCs) resulting in increased expression of CD80 and CD38 (Figure S1). As shown in Figure 1, NSTEMI patients (3.7 ± 0.25) showed increased CD80 expression after LPS stimulation as compared with SA patients (2.58 ± 0.29; *p =* 0.008) and HC (2.45 ± 0.24; *p =* 0.002). No differences were observed in CD38 expression among the three groups of study. 

### 2.2. Altered IDO Induction and Activity in Mmddcs of NSTEMI Patients

**Figure 2:**
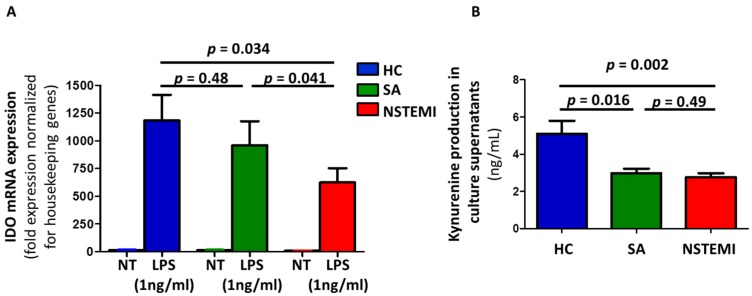
Indoleamine 2,3-dioxygenase (IDO) expression and activity in cultured mMDDCs. Monocytes from 18 NSTEMI, 16 SA and 16 HC were differentiated and activated in vitro to obtain mMDDCs (as described in Figure 1 and Figure S1). (**A**) Stimulation with lipopolysaccharide (LPS) induced the expression of IDO mRNA (assessed by real-time quantitative polymerase chain reaction (RT-qPCR)) in mMDDCs in all three groups of study. Notably, LPS-maturated MDDCs from NSTEMI patients showed lower expression of IDO mRNA compared with SA and HC (*p* for trend < 0.001). Data were normalized to β-2microglobulin or GAPDH as housekeeping genes and were expressed as mRNA fold expression using the formula 2^−ΔΔCt^, where Ct is the threshold cycle. *NT* = untreated MDDC; *LPS* = LPS-maturated MDDCs. (**B**) Supernatants of mMDDCs were tested by ELISA for production of the tryptophan catabolite kynurenine, as an index of IDO activity (*p* for trend = 0.004). Data are expressed as means ± SEM.

Stimulation with LPS induced an increase of IDO mRNA expression in mMDDCs in the three groups of study. Notably, mMDDCs of NSTEMI patients (625.0 ± 128.2) showed reduced mRNA expression of IDO after LPS activation as compared with SA (958.5 ± 218.3; *p* = 0.041) and HC (1183.6 ± 231.6; *p* = 0.034) (Figure 2A).

Kynurenine levels reflect IDO activity, so we evaluated the amount of this catabolite in the supernatants of mMDDCs. ELISA assay revealed a reduced concentration of kynurenine in NSTEMI patients (2.78 ± 0.2) and SA patients (2.98 ± 0.25) as compared with HC (5.1 ± 0.69; *p* = 0.002 and *p* = 0.016, respectively) (Figure 2B).

### 2.3. Altered Th1/Treg Differentiation in NSTEMI and SA Patients

**Figure 3:**
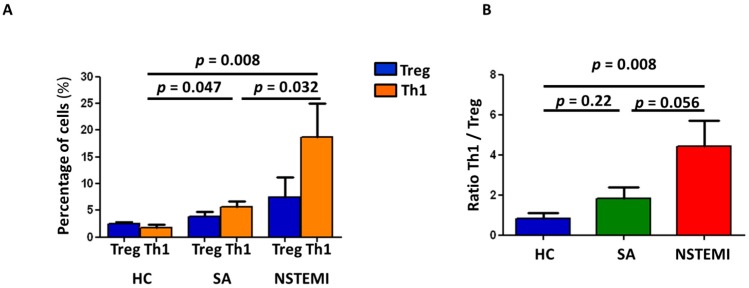
Mature MDDCs promote the differentiation of naïve T-cells. Naïve T-cells obtained from HC, SA and NSTEMI (5 patients for each group) were co-cultured for 6 days with autologous mMDDCs at 10:1 ratio. Afterwards, T-cell differentiation was analyzed by flow-cytometry as described in Figure S2. (**A**) NSTEMI patients showed increased Th1 differentiation compared to HC and SA (*p* for trend = 0.006). No differences in absolute Treg frequency were observed among the three groups of study. (**B**) Histograms show the ratio between the percentages of differentiated Th1/Treg cells. NSTEMI and SA patients have significantly higher ratio favoring Th1 cells compared to HC, with NSTEMI having the higher ratio (*p* for trend = 0.014). Data are expressed as means ± SEM.

To assess if the altered MDDC maturation and IDO pathway could affect T-cell differentiation, we performed co-culture experiments between autologous LPS-maturated MDDCs and isolated naïve CD4^+^ T-cells. As shown in Figure 3A, after six days of co-culture, both NSTEMI (18.7 ± 6.4) and SA (5.6 ± 1.1) patients showed increased Th1 differentiation as compared with HC (1.8 ± 0.5) (*p =* 0.008 and *p* = 0.047, respectively), with NSTEMI patients showing the highest frequency of Th1 expression (NSTEMI vs. SA *p* = 0.032). Moreover, although the percentage of Treg did not significantly differ among the three groups of study, NSTEMI patients (4.4 ± 2.9) showed a higher Th1/Treg ratio as compared with SA patients (1.8 ± 0.6; *p* = 0.056) and HC (0.9 ± 0.3; *p* = 0.008) (Figure 3B).

**Figure 4:**
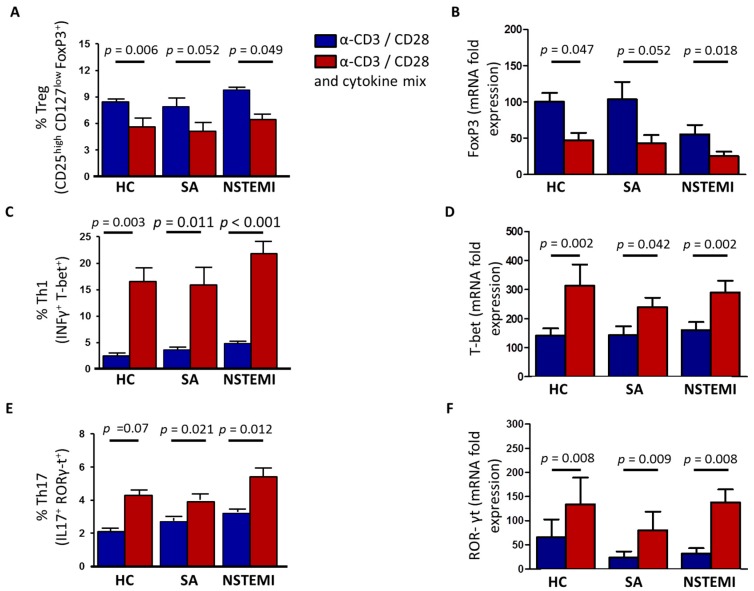
Naïve T-cell differentiation after T-cell receptor (TCR) activation and exposure to cytokine mixture. Naïve CD4^+^ T-cells were stimulated for six days with anti-CD3/-CD28-coated beads alone (blue histograms) or in presence of a cytokine mixture (red histograms) including IL-2, IL-12, IL-1β, IL-6, TGF-β, IL-23, IL-10, anti-human-IL-4. The cytokine cocktail reduced the frequency of Treg as assessed by flow-cytometry (panel (**A**)) and the expression of Foxp3 lineage specific gene (panel (**B**)) and increased the frequency of Th1 and Th17, as assessed by flow-cytometry (panel (**C**,**E**)) and the expression of their lineage specific genes T-bet and Rorγ-t, as assessed by RT-qPCR (panel (**D**,**F**)). No statistical differences were observed among the three groups of study under these experimental conditions. Cumulative data from 10 NSTEMI10 SA and 10 HC are expressed as mean ± SEM. Th1, Th17 and Treg characterization by flow-cytometry has been described in Figure S2.

To investigate whether the increased Th1 differentiation observed in NSTEMI and SA patients was the result of altered MDDCs’ maturation and IDO-dependent immune-modulation or an intrinsic T-cell abnormality, we stimulated TCR of freshly isolated naïve CD4^+^ T-cells from NSTEMI, SA and HC with anti-CD3/-CD28-coated beads. Moreover, to evaluate the role of the cytokine environment, we used a cytokine mixture (containing IL-2, IL-12, IL-1β, IL-6, IL-23, TGF-β, IL-10, anti-human-IL-4). After six days of culture, we assessed the frequency of Th1, Th17 and Treg subsets (Figure S2) and the expression of their lineage specific genes, T-bet, Rorγ-t and Foxp3, respectively. As shown in Figure 4, the exposure to cytokine mixture affected naive T-cell differentiation by increasing the expression of Th1 and Th17 effector cells and reducing the frequency of Treg. However, no differences were observed among the three groups of study under these experimental conditions.

## 3. Discussion

Immune mechanisms involving innate and adaptive immunity play a pivotal role in atherosclerosis [20].

Several studies have shown that in ACS patients, CD4^+^ T-cell subpopulations are dysregulated; in particular, the numbers and the functions of regulatory T cells (Treg) are reduced, while the effector lymphocytes Th1 and Th17 are expanded [2,3,4]. Moreover, the production of pro-inflammatory cytokines is not adequately counterbalanced by anti-inflammatory cytokines, such as IL-10 and TGF-β, and this imbalance has been related to short- and long-term prognosis [21,22,23].

The generation of different T-cell subsets is controlled by cytokine environment, by TCR-mediated signal strength and by antigen presenting cells [1]. The expansion of different T-helper cell subsets will determine the course of the immune response.

In the present study, we observed an alteration in MDDC maturation after ex vivo stimulation with LPS in NSTEMI patients compared with SA and HC. MDDCs from NSTEMI patients showed an increased expression of the co-stimulatory molecule CD80 and a reduced expression of the immunomodulatory enzyme IDO. In NSTEMI and SA patients, we also observed an increase of Th1 differentiation after ex vivo polarization of naïve CD4^+^ T-cells with LPS-maturated MDDCs, but we did not observe any difference in T-cell differentiation after TCR stimulation with anti-CD3^−/−^CD28-coated beads in the presence or absence of a cytokine environment.

DCs are highly specialized APCs with the unique capacity to establish and control immune responses. Activation of DCs is an important initial step in the cascades of events leading to many chronic inflammatory diseases, including atherosclerosis. However, the exact role of DCs in NSTEMI has been poorly investigated.

Maturation induces deep modifications in DCs’ phenotype that are critical for antigen presentation to adaptive immune cells and, thus, for T-cell activation and differentiation. The evidence that MDDCs from NSTEMI showed increased expression of CD80 and reduced expression of IDO suggests that in NSTEMI patients there is an altered DC maturation process. Our observation of maturation abnormalities in DCs from NSTEMI patients is in line with previous studies [24,25]. Notably, we observed an increase in Th1 frequency after DC presentation whereas there were no differences in T-cell differentiation after TCR stimulation and exposure to cytokine mixture, suggesting that the aberrant MDDC maturation might be a relevant mechanism underlying the T-cell dysregulation in NSTEMI patients.

The reduced expression of IDO in MDDCs of NSTEMI patients may exacerbate a chronic inflammatory microenvironment by polarizing the immune response towards effector Th1 lymphocytes.

IDO-induced tryptophan degradation is a widely recognized defense mechanism regulating immunity, resulting in suppression of T-cell activity [13,17] and limiting growth of intracellular pathogens and proliferation of tumor cells [26,27].

Considerable evidence supports the hypothesis that IDO may also play a role in maintaining plaque stability and its metabolites may have beneficial effects on atherosclerosis, inflammation and lipid metabolism. In a murine model of atherosclerosis, IDO deficiency was associated with a significant increase in atherosclerotic lesion size and surrogate markers of plaque vulnerability, through downregulation of IL-10 production. Administration of 3,4-dimethoxycinnamoyl anthranilic acid (3,4-DAA), an orally active synthetic derivate of the tryptophan catabolite anthranilic acid, was associated to a significant reduction in lesion size and inflammation [28]. Furthermore, in ApoE^−/−^ mice, administration of 1-methyl tryptophan (1-MT), a systemic IDO inhibitor, led to a significant increase in atherosclerotic lesions and enhanced vascular inflammation, by the up-regulation of vascular cell adhesion molecule-1 (VCAM-1) and monocyte chemoattractant protein-1 (MCP-1/CCL2) leading to increased macrophage infiltration into the plaque. This IDO deficiency-induced accelerated atherosclerosis could be reverted by exogenous administration of the tryptophan metabolite 3-hydroxykynurenine, 3-hydroxyanthranilic acid (3-HAA) [29]. In line with this, 8 week treatment with IDO catabolite 3-HAA in atherosclerosis prone LDLr^−/−^ mice has been associated to a significant reduction in lesion size, through inhibition of ox-LDL uptake by macrophages, decrease of CD4^+^ T-cells infiltration in atherosclerotic lesions and reduction in serum cholesterol and triglyceride levels [30]. Moreover, plasmacytoid DCs seem to have a protective role in a mouse model of atherosclerosis, by dampening T-cell proliferation and function in an IDO-dependent manner [31].

### Study Limitations

Our study was an observational analysis that included a limited number of patients. No power calculation could be performed because of a lack of previous studies in this setting. Moreover, our ex vivo experiments were performed in MDDCs and might not reflect the histological features of the atherosclerotic plaque (since we do not have histological specimens of our patients), or the biological outcome of the physiological DCs. In our study, we used strong stimuli for T-cell activation and MDDC differentiation (MDDC LPS-stimulated and anti-CD3/-CD28-coated-beads). Further and deeper studies will help to determine the role of the strength of TCR activation in NSTEMI naïve T-cell differentiation.

## 4. Materials and Methods

### 4.1. NSTEMI and SA Patients and Healthy Subjects Enrolled in the Study

We enrolled 37 patients admitted to our coronary care unit with a diagnosis of non-ST elevation myocardial infarction (NSTEMI), defined as detection of rise and fall of cardiac troponin I (cTnI) and at least one of the following: angina, ST-segment depression or T-wave inversion. As control groups, we enrolled 27 patients with chronic stable angina (SA) admitted to our cardiovascular ward to undergo coronary angiography because of severe symptoms (CCS class III or IV) and/or high-risk abnormalities on non-invasive testing, and 22 subjects aged >50 years without overt cardiovascular diseases (HC).

Patients enrolled in the SA group had symptoms of stable effort angina lasting more than 12 months, angiographically confirmed coronary artery disease, no previous NSTEMI and no overt ischemic episodes during the previous 48 h.

HC were screened in our out-patients clinic among subjects at intermediate risk for cardiovascular diseases. A complete cardiovascular screening was performed, including a standard 12-lead EKG, a treadmill EKG stress test, an echocardiogram, an echo-colordoppler of carotid arteries.

Exclusion criteria were: (1) age > 80 years; (2) evidence of inflammatory or infectious diseases, malignancies, immunologic or haematological disorders; (3) allergic disorders; (4) ejection fraction <40%; (5) treatment with anti-inflammatory drugs other than low-dose aspirin. Demographic data, classical cardiovascular risk factors, history of previous NSTEMI, previous coronary revascularization procedures, ventricular function and medical treatment were evaluated (Table 1). All NSTEMI and SA patients underwent coronary angiography; in NSTEMI, coronary angiography was performed within 72 h after admission; in-hospital revascularization procedures were recorded. All patients gave their written informed consent. The Ethics Committee of the Catholic University of the Sacred heart of Rome approved the study (R4124500186), on 5 May 2014.

Venous blood samples were obtained at the time of hospital admission in NSTEMI and SA patients and at the out-patient visit in HC. In four NSTEMI patients, we did not obtain enough mononuclear cells from the blood samples to purify a sufficient number of monocytes for our experiments. Thus, in these patients we did not perform any experimental analysis, although we maintained them as part of the study population. 

We evaluated CD80 expression (Figure 1), IDO mRNA expression (Figure 2A) and kynurenine catabolite levels (Figure 2B) on monocytes isolated from 18 out of the remaining 33 NSTEMI patients. The same experiments were performed on monocytes isolated from 16 SA patients and 16 HC subjects.

Co-culture experiments between autologous LPS-maturated MDDCs and isolated naïve CD4+ T-cells were performed on cells isolated from 5 patients of each group who were not included in the previous experiments (Figure 3).

Naïve T-cell differentiation after TCR activation and exposure to cytokine mixture were performed on cells isolated from 10 patients of each group: all 10 NSTEMI patients were not included in the previous experiments; 4 SA patients and 9 HC subjects were already included in previous experiments.

### 4.2. Cell Isolation and Cultures

Peripheral blood mononuclear cells (PBMCs) were enriched from whole blood by density centrifugation over Ficoll-Hypaque (GE Healthcare Bio-Sciences, Piscataway, NJ, USA). Highly enriched monocytes CD14^+^ cells were purified from total PBMCs by magnetic cell sorting (CD14^+^ cell isolation kit, MiltenyiBiotec, Auburn, CA, USA), typically resulting in a greater than 95% enrichment of targeted cell population. Purity and viability were monitored by flow cytometry. These monocyte-enriched cell populations were cultured in complete RPMI 1640 medium (Sigma, St. Louis, MO, USA) with 10% fetal bovine serum (Invitrogen, Carlsbad, CA, USA) supplemented with 25 ng/mL GM-CSF (Miltenyi Biotech, Auburn, CA, USA) and 25 ng/mL IL-4 (Miltenyi Biotech, Auburn, CA, USA) for 6 days under sterile conditions at 37 °C in an atmosphere containing 5% CO_2_, to generate iMDDCs. MDDC differentiation was assessed measuring the expression of CD11c and CD14 by flow cytometry. MDDCs were used in the experiments if >90% CD11c and <5% CD14.

For MDDC activation, iMDDC were exposed to 1 ng/mL LPS (Sigma, St. Louis, MO, USA) for 24 h, as previously described [32]. MDDC maturation was assessed measuring the expression of CD80 and CD38 (Figure S1).

Highly enriched naïve CD4^+^CD45RA^+^ T-cells were isolated from total PBMCs by magnetic cell sorting (CD4^+^ Naïve T-cell isolation kit II, human, MiltenyiBiotec, Auburn, CA, USA) with a purity >95% (Figure S2). Naïve CD4^+^ T cells were cultured at a density of 10^6^ cells/mL in complete RPMI 1640 medium and activated with αCD3/-CD28-coated beads (Dynabeads, T cell activators, Life Technologies, Carlsbad, CA, USA) at a Tcell:microbead 1:1 ratio with or without a cytokines milieu including IL-2 (30 U/mL, Thermo Scientific, Waltham, MA, USA), IL-12 (2 ng/mL, Miltenyi Biotech, Auburn, CA, USA), IL-1β (10 ng/mL, Thermo Scientific, Waltham, MA, USA), IL-6 (10 ng/mL, Thermo Scientific, Waltham, MA, USA), TGF-β (10 ng/mL, Thermo Scientific, Waltham, MA, USA), IL-23 (25 ng/mL, eBioscience, San Diego, CA, USA), IL-10 (10 ng/mL, Meridian, Life Science, Memphis, TN, USA), anti-human-IL-4 (10 µg/mL, eBioscience, San Diego, CA, USA).

### 4.3. Mixed Lymphocyte Reaction

Freshly purified CD4^+^CD45RA^+^ T-cells were cryo-preserved in 90% fetal bovine serum (Invitrogen, Carlsbad, CA, USA) +10% DMSO for 6 days. Defrosted naïve CD4^+^ T-cells were co-cultured under sterile conditions at 37 °C in an atmosphere containing 5% CO_2_ with autologous MDDCs at a Tcell:DC ratio of 10:1 for 6 days and IL-2 was added the third day for the expansion of differentiated T-cells.

### 4.4. Flow Cytometric Analysis

Purity of CD4^+^ naïve T-cell preparations was assessed by cytofluorimetric staining: anti-CD4-FITC, anti-CD45RA-ECD and anti-CCR7-PE (Beckman Coulter, Brea, CA, USA). Immature DCs were identified as CD14^−^CD11c^+^ CD80^low/null^, while mature DCs were CD11c^low^ CD80^high^ using anti-CD11c-FITC (Miltenyi Biotech, Auburn, CA, USA), anti-CD14-ECD, anti-CD80-PE (Beckman Coulter, Brea, CA, USA) (Figure S1). Differentiated Th1, Th17 and Treg from naïve CD4^+^ T-cells were characterized as described in Supplementary Figure 2 using anti-CD25-PC5, anti-CD127-PC7 (Beckman Coulter, Brea, CA, USA) and anti-Foxp3-PE, anti-T-bet-PE, anti-IFNγ-PC7, anti-RORγt-PE and anti-IL17-FITC (eBioscience, San Diego, CA, USA).

Cytokine production by CD4^+^ T-cell subset was assessed after 4 h in vitro stimulation with 100 ng/mL phorbol-2-myristate-13-acetate (PMA) (Sigma, St. Louis, MO, USA) and 1 µg/mL ionomycin (Sigma, St. Louis, MO, USA). Cells were incubated at 37 °C for a total of 4 h; during the last 2 h, 10 µg/mL brefeldin A (Sigma, St. Louis, MO, USA) was added to block extracellular secretion of cytokines.

For the detection of intracellular factors, cells were fixed and permeabilized with Fix/Perm buffer (eBioscience, San Diego, CA, USA) and stained with specific antibodies. Analyses of stained cells were performed using a FC500 flow cytometry system (Beckman Coulter, Brea, CA, USA), and Kaluza^®^ analysis software packages (Beckman Coulter, Brea, CA, USA) was used for data analysis.

### 4.5. RNA Extraction

CD4^+^ T-cells and MDDC total RNA was isolated using RNeasy Plus Mini Kit (Qiagen GmbH, Hilden, Germany) according to the manufacture instruction. Then, 250 ng and 500 ng RNA respectively from CD4^+^ T-cells and DCs were reverse transcribed into cDNA using iScript RT (Bio-Rad laboratories, Hercules, CA, USA).

### 4.6. Real Time Quantitative Polymerase Chain Reaction (RT-qPCR)

RT-qPCR was performed with iQ5 Multicolorreal time PCR detection system (Bio-Rad laboratories, Hercules, CA, USA) using iQ SYBR Green Supermix (Bio-Rad laboratories, Hercules, CA, USA). Oligonucleotide primers for IDO (For 5′-TCATCTCACAGACCACAA-3′, Rev 5′-GCAGTAAGGAACAGCAATA-3′), Foxp3 (For 5′-GAGAGGTCTGCGGCTTCCAC-3′, Rev 5′-GGGCATCGGGTCCTTGTCC-3′), T-bet (For 5′-TGTGACCCAGATGATTGT-3′, Rev 5′-AAGATATGCGTGTTGGAAG-3′), ROR-γt (For 5′-TGAGAACACAAATTGAAGTGA-3′, Rev 5′-CAGGTGATAACCCCGTAG-3′), β-2 microglobulin (For 5′-AGGACTGGTCTTTCTATCTCTTGT-3′; Rev 5′-ACCTCCATGATGCTGCTTACA-3′) and GAPDH (For 5′-CAACAGCCTCAAGATCATCAG-3′, Rev 5′-GAGTCCTTCCACGATACCA-3′) were designed using the software Beacon Design. Then, 1 µL of cDNA was used as template for RT-qPCR in a 15 µL reaction volume on triplicate samples. Data were normalized to β-2microglobulin or GAPDH as housekeeping genes and were expressed as mRNA fold expression using the formula 2^−ΔΔCt^, where Ct is the threshold cycle.

### 4.7. Kynurenine Production 

The kynurenine production was assessed by human kynurenine ELISA kit (BlueGene Biotech, Shanghai, China). After MDDC maturation, supernatants were collected. Supernatant samples were stored at −80 °C until use and thawed on ice the day of the assays. To remove any precipitate, samples were centrifuged for 15 m at 3000 r.p.m. Each sample was run in duplicate according to the manufacturer’s instructions using the provided reagents.

### 4.8. Statistical Analysis 

One-way ANOVA for repeated measures, with Bonferroni correction, was used for multiple pairwise comparisons and paired-samples *t*-test to compare the means of two related samples within groups. A two-tailed *p*-value < 0.05 was considered statistically significant. Statistical analysis was performed with GraphPad Prism version 5.00 for Windows (GraphPad Software, San Diego, CA, USA) and SPSS 18.0 software (SPSS Inc., Chicago, IL, USA). Multivariate logistic regression analysis was applied to individuate the variables independently associated with the phenotype of mMDDC (CD80 and IDO expression). Only variables with a value of *p* ≤ 0.05 at univariate analysis were included in the multivariate model, with age and sex as confounding variables (Tables S1A and S1B).

## 5. Conclusions

In NSTEMI patients, the tolerogenic mechanism of the immune response related to IDO production by ex vivo activated MDDCs is altered, supporting its role in T-cell dysregulation observed in this clinical setting. Understanding the signaling pathway responsible for MDDC maturation abnormalities might pave the way to the identification of novel molecular targets in the subset of NSTEMI patients in whom uncontrolled immune activation impairs coronary plaque stability.

## Figures and Tables

**Table 1:** Summary of the the clinical characteristics of NSTEMI and stable angina (SA) patients and healthy subjects enrolled in the study.

	HC	SA	NSTEMI	*p*-Value
Number	22	27	37	
Sex (M/F)	14/8	22/5	33/4	0.06
Age (mean ± SD)	64 ± 27	47 ± 32	66 ± 11	0.74
RISK FACTORS				
Hypercholesterolemia, *n* (%)	10 (45)	13 (35)	15 (41)	0.82
Hypertension, *n* (%)	12 (54)	19 (70)	31 (83)	0.051
Smoking habit, *n* (%)	3 (14)	17 (63)	21 (57)	<0.001
Family History of IHD, *n* (%)	5 (23)	2 (7)	14 (38)	0.020
Diabetes, *n* (%)	5 (23)	10 (37)	7 (19)	0.24
Previous History				
NSTEMI, *n* (%)	NA	NA	9 (24)	-
Previous PCI/CABG, *n* (%)	NA	NA	9 (24)	-
Medications (at the time of blood sampling)				
Aspirin, *n* (%)	2 (10)	8 (30)	17 (46)	0.013
Ticlopidin/Clopidogrel, *n* (%)	1 (5)	3 (11)	7 (19)	0.27
β-blockers, *n* (%)	3 (14)	6 (22)	12 (32)	0.26
ACE-inhibitors/ARBs, *n* (%)	4 (18)	8 (30)	15 (41)	0.20
Statins, *n* (%)	6 (27)	8 (30)	16 (43)	0.37
Insulin, *n* (%)	2 (10)	3 (11)	3 (8)	0.73
Oral antidiabetic drugs, *n* (%)	3 (14)	7 (26)	4 (11)	0.71
In-hospital Management				
cTnI > 0.01 ng/mL, *n* (%)	0	0	37 (100)	-
Multi-vessel disease, *n* (%)	0	13 (48)	17 (46)	0.86
PCI/CABG for the index event, *n* (%)	0	13 (48)	28 (76)	<0.001
Laboratory Assay (mean ± SD)				
Total Cholesterol (mg/dL)	201 ± 40.9	193.8 ± 40.5	188 ± 36.6	0.58
LDL (mg/dL)	104 ± 37.1	107.4 ± 41.3	115 ± 35.28	0.42
HDL (mg/dL)	54.14 ± 13.3	50.8 ± 12.4	45.2 ± 11.2	0.063
Triglycerides (mg/dL)	117.9 ± 59.5	10^9^.1 ± 40.6	149.6 ± 58.3	0.065
WBC (10^9^/L)	8.27 ± 2.5	7.59 ± 2.56	8.74 ± 3.5	0.63
Lymphocytes (10^9^/L)	1.82 ± 0.6	1.84 ± 0.4	1.44 ± 0.6	0.49
Neutrophil (10^9^/L)	6.43 ± 3.12	4.69 ± 1.36	5.83 ± 2.86	0.14
Monocytes (10^9^/L)	0.54 ± 0.26	0.44 ± 0.14	0.46 ± 0.20	0.20

HC = healthy controls; SA = stable angina; NSTEMI = non-ST elevation myocardial infarction; IHD = ischemic heart disease; PCI = percutaneous coronary intervention; CABG = coronary artery by-pass surgery; ARBs = Angiotensin II receptor blockers; TnI = troponin I. *p*-values refer to general differences between groups (ANOVA).

## Supplementary Materials

The following are available online at www.mdpi.com/1422-0067/19/1/63/s1.
