# Supplementary material for: Indoleamine 2,3-Dioxygenase (IDO) Enzyme Links Innate Immunity and Altered T-Cell Differentiation in Non-ST Segment Elevation Acute Coronary Syndrome
Source: Int J Mol Sci. 2017 Dec 26;19(1):63. doi: 10.3390/ijms19010063 (PMC5796013; doi:10.3390/ijms19010063)
Supplement: Supplementary file 1 [file ijms-19-00063-s001.pdf]

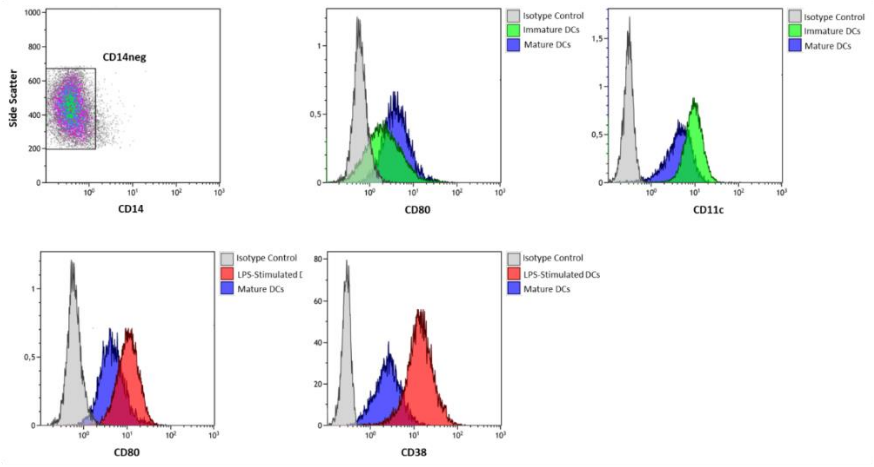

Supplementary Figure 1. ***Upper panels***: Immature MDDCs were identified as CD14$^-$CD11c$^+$ CD80$^{low/null}$ (green histogram) while mature MDDCs were CD11c^{low} CD80^{high} (blue histogram). ***Lower panel***: MDDCs maturation was assessed measuring the expression of CD80 and CD38.

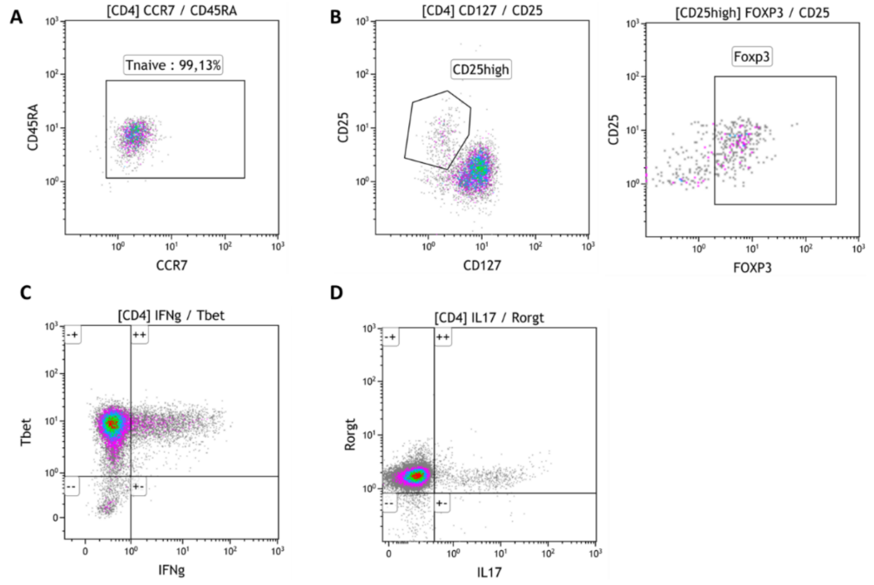

Supplementary Figure 2. (**A**) Highly enriched naïve CD4$^+$ purity was assessed using CD45RA and CCR7 antibody. (**B**) Treg were identified as CD25^{high}CD127^{low}Foxp3$^+$. (**C**) Th1 cells were identified as Tbet$^+$IFNγ^+. (**D**) Th17 cells were identified as Rorγt$^+$IL17$^+$.

Supplementary Table 1A. Univariate and multivariate logistic regression analysis to individuate the variables independently associated with CD80 expression on mMDDCs.

	**Univariate Analysis**		**Multivariate Analysis**	
	**HR (CI 95%)**	***p***	**HR (CI 95%)**	***p***
Age	1.05 (1.01–1.09)	0.008	1.02 (0.96–1.08)	0.44
Sex	0.45 (0.18–1.08)	0.07	1.46 (0.44–4.9)	0.53
NSTEMI diagnosis	2.61 (1.21–5.65)	0.001	2.28 (0.79–6.57)	0.02 *
Familial History of IHD	0.72 (0.33–1.56)	0.4		
Hypertension	1.57 (0.74–3.33)	0.24		
Hypercolesterolemia	1.16 (0.55–2.45)	0.7		
Smoke	1.14 (0.53–2.41)	0.7		

Diabetes	2.61 (0.6–10.4)	0.17		
Statin treatment	1.11 (1.02–1.21)	0.01	1.08 (0.98–1.21)	0.11
ACE-inhibitor/ARB treatment	0.80 (0.24–2.68)	0.73		
Oral antidiabetics/insulin treatment	0.97(0.86–1.09)	0.63		
Acetylsalicylic acid treatment	0.76 (0.20–2.78)	0.67		

The analysis included 18 NSTEMI patients, 16 SA patients and 16 HC subjects. **IHD** = ischemic heart disease; **ARBs** = Angiotensin II receptor blockers. * Only the diagnosis of NSTEMI at the time of study enrollment was independently associated with CD80 expression on mMDDCs.

Supplementary Table 1B. Univariate and multivariate logistic regression analysis to individuate the variables independently associated with IDO mRNA expression in mMDDCs.

	**Univariate Analysis**		**Multivariate Analysis**	
	**HR (CI 95%)**	***p***	**HR (CI 95%)**	***p***
Age	1.02 (0.96–1.07)	0.48	1.00 (0.94–1.07)	0.85
Sex	0.57 (0.18–1.77)	0.3	0.76 (0.20–2.78)	0.67
NSTEMI diagnosis	5.6 (2.24–14.9)	0.001	3.32 (1.02–10.8)	0.04*
Familial History of IHD	1.3 (0.27–6.19)	0.7		
Hypertension	0.5 (0.16–1.57)	0.23		
Hypercolesterolemia	1.35 (0.33–5.42)	0.6		
Smoke	0.87 (0.27–2.75)	0.81		
Diabetes	2.8 (0.96–8.13)	0.06		
Statin treatment	2.8 (0.96–8.13)	0.05	2.0 (0.6–6.9)	0.26
ACE-inhibitor/ARB treatment	1.00 (0.88–1.13)	0.94		
Oral antidiabetics/insulin treatment	1.02 (0.96–1.07)	0.48		
Acetylsalicylic acid treatment	0.57 (0.18–1.77)	0.3		

The analysis included 18 NSTEMI patients, 16 SA patients and 16 HC subjects. **IHD** = ischemic heart disease; **ARBs** = Angiotensin II receptor blockers. * Only the diagnosis of NSTEMI at the time of study enrolment was independently associated with IDO mRNA expression in mMDDCs.
